# Quantitative Description of Osteopathic Physician Authorship in Prominent Neurosurgery Journals Since 1944: Coming of Age?

**DOI:** 10.7759/cureus.3124

**Published:** 2018-08-09

**Authors:** Joshua A Cuoco, Christopher M Busch, Cara M Rogers, Evin L Guilliams, Brendan J Klein, Gregory A Howes, Eric A Marvin

**Affiliations:** 1 Neurosurgery, Carilion Clinic - Virginia Tech Carilion School of Medicine, Roanoke, USA; 2 Neurosurgery, Carilion Clinic - Virginia Tech Carilion School of Medicine, Roanoke , USA

**Keywords:** academic medicine, medical education, osteopathic physician, allopathic physician, neurosurgery

## Abstract

Background

The Accreditation Council for Graduate Medical Education and the American Osteopathic Association recently agreed to establish a single graduate medical education system for the United States allopathic and osteopathic resident physicians by 2020. Consequential to this merger, new standards will be implemented for academic and research requirements within medical schools as well as residency programs. In the United States, osteopathic medicine is considered to be a parallel profession to allopathic medicine. However, recent studies have revealed that the percentages of United States osteopathic physicians currently in practice are not proportional to the percentages of editorial board member positions they hold in several high-profile medical journals as well as neurosurgical journals. To our knowledge, there is currently no published literature examining osteopathic physician author representation of any neurosurgical journal. In the present study, we analyze the number of osteopathic physicians and osteopathic neurosurgeons serving as authors in prominent neurosurgical journals.

Methods

American neurosurgical journals with the highest number of citations plus an affiliation with a neurosurgical society open to osteopathic neurosurgeons were used as criteria for journal selection. The Journal of Neurosurgery Publishing Group journals (*Journal of Neurosurgery, Journal of Neurosurgery: Spine, Journal of Neurosurgery: Pediatrics*, and *Neurosurgical Focus*) fulfilled these criteria. The number of allopathic and osteopathic physicians who have published at least one manuscript in a Journal of Neurosurgery Publishing Group journal was counted. The specialty of each osteopathic author was examined.

Results

Our analysis found that allopathic physicians represented 105,157 (99.68%) and osteopathic physicians represented 335 (0.32%) of the 105,492 authorship positions held by these physicians in these journals since 1944. Statistical significance was found comparing the number of allopathic authors versus the number of osteopathic authors (p < 0.0001). The most common specialty represented by osteopathic authors in all journals was neurosurgery (45%). Osteopathic neurosurgeons represented 153 (0.15%) of the total number of allopathic and osteopathic authors.

Conclusions

These data establish that the percentages of the United States osteopathic physicians and osteopathic neurosurgeons currently in practice are not proportional to the percentages of authorship positions they hold in Journal of Neurosurgery Publishing Group journals. We postulate that this apparent disproportionality may originate from significant differences between allopathic and osteopathic medical school research funding, research opportunities, scholarly activities, and dual-degree programs.

## Introduction

Osteopathic medicine was founded more than 125 years ago in Kirksville, Missouri. Since then, the number of practicing osteopathic physicians in the United States (US) has increased considerably. In 2016, 67,534 (7.7%) of the active US physician population were osteopathic physicians, 586,287 (66.8%) were US-trained allopathic physicians, and 214,681 (24.5%) were international medical graduates [1]. With nearly one out of four US medical students currently enrolled in an osteopathic medical school, osteopathic medicine is on the rise [2]. Moreover, the Accreditation Council for Graduate Medical Education (ACGME) and the American Osteopathic Association (AOA) recently agreed to establish a single graduate medical education system for the United States allopathic and osteopathic residents by 2020, which will establish new academic and research requirements for medical schools and residency programs. Although 7.7% of the US physician population currently holds an osteopathic medical degree, data published by Ashurst and Galuska indicated that osteopathic physicians occupied 0.15% of 2058 editorial positions in eight major medical journals [1,3]. Hoehmann et al. demonstrated that osteopathic physicians comprised 0.14% of 2826 editorial positions in 50 continuing neurosurgery journals [4]. Furthermore, osteopathic neurosurgeons comprise 1.7% of the US neurosurgery population yet Hoehmann et al. observed that they only constitute 0.04% of editorial positions in current neurosurgery journals [1,4]. To our knowledge, there is currently no literature examining allopathic and osteopathic physician author representation of any neurosurgical journal. In the present study, we investigate the number of allopathic and osteopathic physicians serving as authors in prominent neurosurgery journals as well as analyze the specialty of each osteopathic author represented. We examine the current medical education literature emphasizing the differences between allopathic and osteopathic medical school research funding, research opportunities, and scholarly activities. Moreover, we compare the research experiences and scholarly activities between ACGME and AOA neurosurgery residency applicants.

## Materials and methods

American neurosurgical journals with the highest number of citations as per Thomson Reuters’ Journal Citations Reports plus an affiliation with a neurosurgical society open to osteopathic neurosurgeons were used as criteria for journal selection. The *Journal of Neurosurgery* fulfilled both criteria. However, we included all Journal of Neurosurgery Publishing Group (JNPG) journals (*Journal of Neurosurgery*, *Journal of Neurosurgery: Spine*, *Journal of Neurosurgery: Pediatrics*, and *Neurosurgical Focus*) to broaden our scope of analysis to four journals. Beginning with the inaugural issue of each journal, allopathic and osteopathic physicians who have published at least one manuscript in a JNPG journal were counted. This analysis included each monthly issue in *Journal of Neurosurgery *from 1944 to May 2018, *Neurosurgical Focus* from 1996 to May 2018, *Journal of Neurosurgery: Spine* from 1999 to May 2018, and *Journal of Neurosurgery: Pediatrics *from 2004 to May 2018. Authors’ positions on manuscripts (e.g., first author versus last author) were not specified. An Internet search was performed to determine the medical specialty of each author holding an osteopathic degree. The specialties of osteopathic authors were analyzed. Descriptive statistics were utilized on the collected data to determine the number of allopathic and osteopathic physicians serving as authors in each of the four journals. A paired *t* test was then performed to compare the number of allopathic authors versus the number of osteopathic authors.

## Results

Our analysis found that 105,492 allopathic or osteopathic physicians have published at least one manuscript in a JNPG journal (*Journal of Neurosurgery, Journal of Neurosurgery: Spine, Journal of Neurosurgery: Pediatrics,* or *Neurosurgical Focus*). Allopathic physicians represented 105,157 (99.68%) and osteopathic physicians represented 335 (0.32%) of the 105,492 authorship positions held by these physicians. Of the 105,492 positions, osteopathic physicians accounted for 89 (0.14%) of the total number of authors in *Journal of Neurosurgery*, 86 (0.53%) of the authors in *Journal of Neurosurgery: Spine*, 105 (0.87%) of the authors in *Journal of Neurosurgery: Pediatrics*, and 55 (0.45%) of the authors in *Neurosurgical Focus* (Table 1). Statistical significance was found comparing the number of allopathic physicians versus the number of osteopathic physicians holding authorship (p < 0.0001).

**Table 1:** Author positions held by allopathic and osteopathic physicians in Journal of Neurosurgery Publishing Group journals

Journal	Allopathic Authors	Osteopathic Authors	Osteopathic Neurosurgeons	Combined Authors
Journal of Neurosurgery	64799 (99.86%)	89 (0.14%)	26 (0.04%)	64888
Journal of Neurosurgery: Spine	16215 (99.47%)	86 (0.53%)	32 (0.20%)	16301
Journal of Neurosurgery: Pediatrics	11914 (99.13%)	105 (0.87%)	68 (0.57%)	12019
Neurosurgical Focus	12229 (99.55%)	55 (0.45%)	27 (0.22%)	12284
Total	105157 (99.68%)	335 (0.32%)	153 (0.15%)	105492

**Figure 1:**
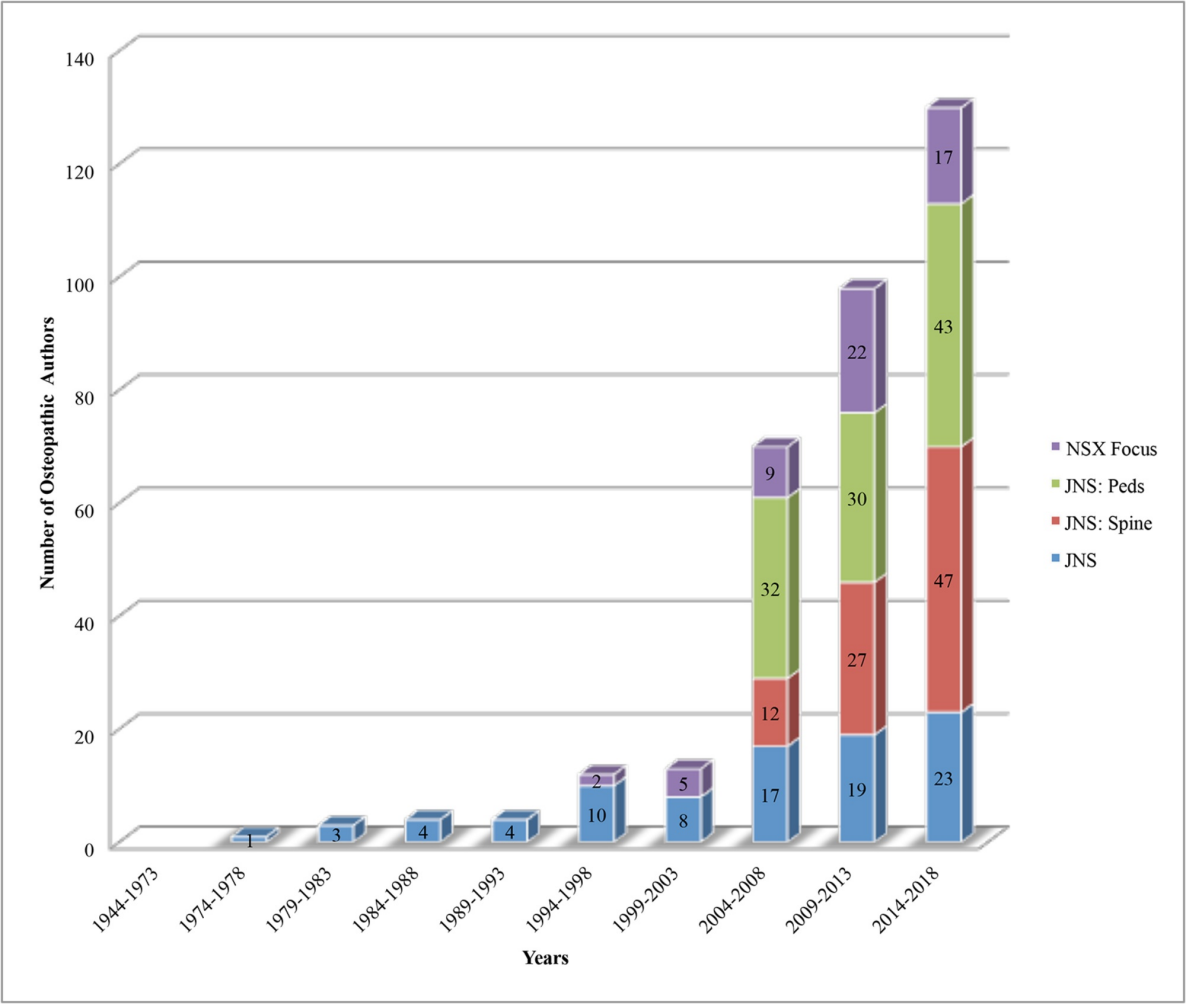
Osteopathic author representation in Journal of Neurosurgery Publishing Group journals since 1944

The first osteopathic physician to serve as an author in a JNPG journal was in 1978 in *Journal of Neurosurgery* (inaugural issue 1944). Since the inception of each journal, we found that representation of osteopathic physician authorship has steadily increased (Figure 1). For example, from 1974 to 1978, one osteopathic physician served as author to *Journal of Neurosurgery* compared to 23 from 2014 to 2018. From 1999 to 2003 (inaugural issue in 1996), five osteopathic physicians served as authors to *Neurosurgical Focus* compared to 17 from 2014 to 2018. From 1999 to 2003 (inaugural issue 1999), zero osteopathic physicians served as authors to *Journal of Neurosurgery: Spine* compared to 47 from 2014 to 2018. From 2004 to 2008 (inaugural issue 2004), 32 osteopathic physicians served as authors to *Journal of Neurosurgery: Pediatrics* compared to 43 from 2014 to 2018.

**Figure 2:**
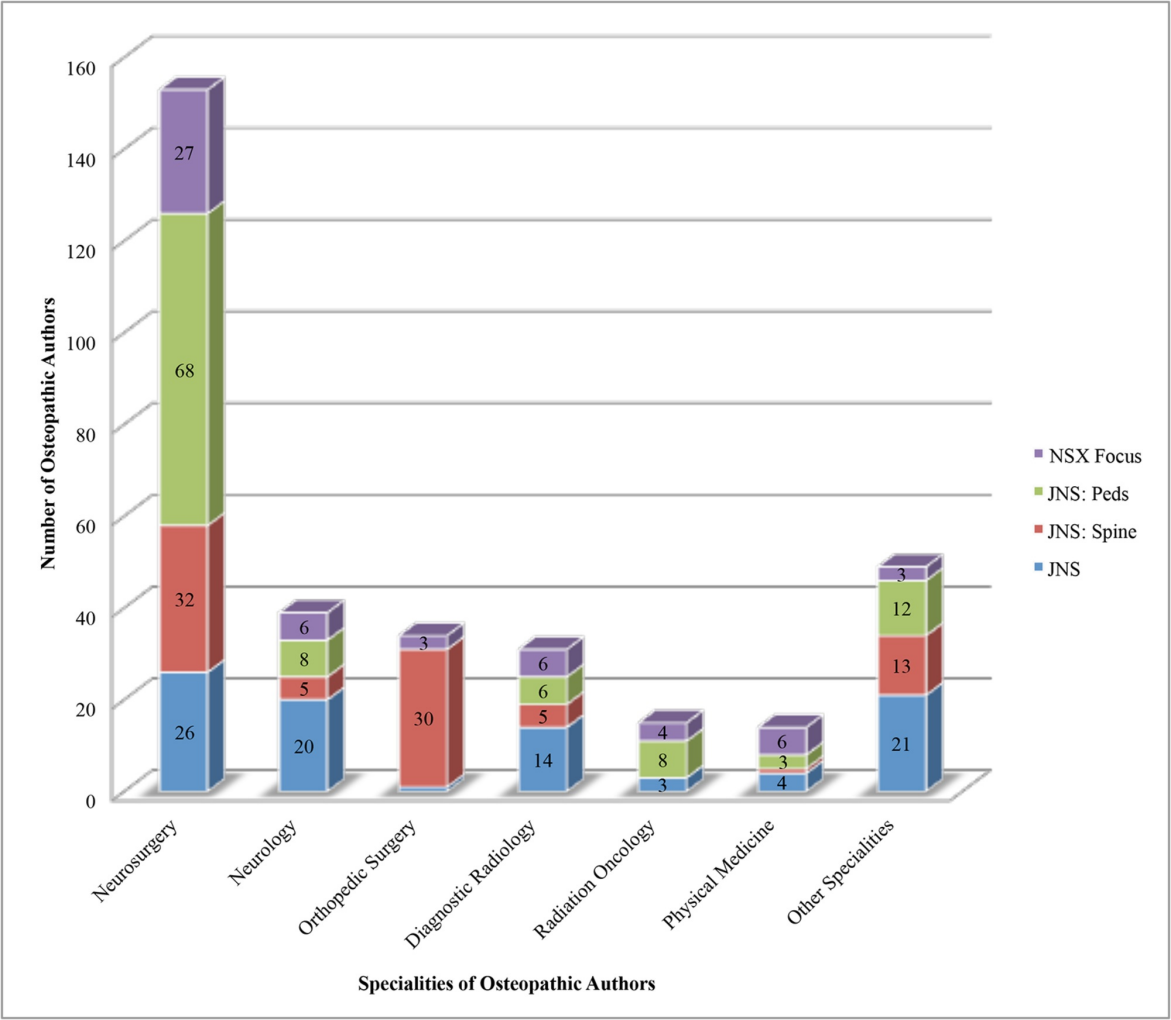
Medical specialties represented by osteopathic authors in Journal of Neurosurgery Publishing Group journals

We then sought to examine the medical specialty of each osteopathic physician serving as author in each journal. The most common specialty represented by osteopathic authors was neurosurgery. Osteopathic neurosurgeons comprised 26 (0.04%) of the combined osteopathic and allopathic authors in *Journal of Neurosurgery*, 32 (0.20%) of authors in *Journal of Neurosurgery: Spine*, 68 (0.57%) of authors in *Journal of Neurosurgery: Pediatrics*, and 27 (0.22%) of authors in *Neurosurgical Focus*. Collectively, the top specialties of osteopathic authors represented in the journals included neurosurgery, neurology, orthopedic surgery, diagnostic radiology, radiation oncology, and physical medicine (Figure 2). The percentage of each osteopathic author specialty represented was calculated. Combining data from these journals, neurosurgery constituted 46% of osteopathic authors, neurology 12%, orthopedic surgery 10%, diagnostic radiology 9%, radiation oncology 4%, and physical medicine 4%. Although neurosurgery was the most common specialty of osteopathic authors in all journals, *Journal of Neurosurgery: Pediatrics* was the only journal where osteopathic neurosurgeons represented greater than 50% of osteopathic authors (65%). Comparatively, osteopathic neurosurgeons constituted 29% of osteopathic authors in *Journal of Neurosurgery*, 37% in *Journal of Neurosurgery: Spine*, and 49% in *Neurosurgical Focus*.

## Discussion

To our knowledge, the present study is the first to analyze osteopathic physician author representation of any neurosurgical journal. Although osteopathic physicians constitute 7.7% of the active US physician population, we report that they only comprise 335 (0.32%) of 105,492 authorship positions held by allopathic and osteopathic physicians in JNPG journals [1]. As expected, neurosurgery was the most common author specialty in all four journals representing 45% of osteopathic authors. However, despite osteopathic neurosurgeons comprising 1.7% of the US neurosurgeon population, we found that they only represented 153 (0.15%) of 105,492 author positions [1]. This study establishes a lack of proportionality between the percentages of osteopathic physicians and osteopathic neurosurgeons currently in practice and the authorship positions they hold in JNPG journals.

The underrepresentation of osteopathic physician and osteopathic neurosurgeon authorship in these journals may be attributed to a multitude of factors beginning with significant differences between allopathic and osteopathic medical school research funding, scholarly activity, and dual-degree programs. Compared to their allopathic counterparts, osteopathic medical school funding from the National Institute of Health (NIH) is insignificant. Indeed, allopathic medical schools received 800 times more funding from the NIH than osteopathic medical schools in 2011 [5]. Furthermore, in the fiscal year 2011, osteopathic medical schools ranked last among 17 types of educational institutions receiving NIH funding [5]. Osteopathic medical school NIH funding lagged behind allopathic medical schools, schools of arts and sciences, public health, engineering, dentistry, pharmacy, veterinary medicine, nursing, social work, education, and optometry [5].

With inadequate research opportunities for medical students to take part in, student’s residency applications may become deficient in scholarly activities – a key factor for resident selection. From 2006 to 2010, 28 osteopathic medical schools collectively published 1843 manuscripts: approximately 13 publications per year per school [5]. Students of osteopathic medical schools have noticed the relative lack of research funding and low quantity of annual publications per school. In fact, a survey of the class of 2017 indicated that 47% of osteopathic seniors felt an insufficient amount of time was devoted within their curriculum to learning research techniques [6]. Moreover, 39% and 37% felt that an insufficient amount of time was devoted to teaching literature analysis and biostatistics, respectively [6]. The same survey reported that graduating students dedicated only 3% of their time during their 3rd and 4th years of medical school to research projects [6]. Lastly, there are fewer osteopathic medical students graduating with a dual-degree (e.g., PhD, MPH, MBA) compared to their allopathic counterparts [7]. Dual-degree PhD programs allow medical students to spend 3-4 additional years in the basic science research laboratory, developing research skills, presenting at conferences, publishing in academic journals, and, ultimately, fostering the making of proficient physician scientists.

Medical school NIH funding translates into medical student research opportunities, scholarly activities, and the beginnings of a career in academic medicine. The absence of a thriving research environment encountered in osteopathic medical schools may impede the development of academic osteopathic physicians and, therefore, constrain osteopathic physicians from publishing during their career. Collectively, these data not only signify that nearly half of osteopathic medical students are not content with the research experiences (or their lack of) offered by osteopathic medical schools but also provide a foundational basis for the lack of proportionality between the percentages of practicing osteopathic physicians and osteopathic neurosurgeons and authorship positions they hold in JNPG journals.

Neurosurgery was the most common specialty represented by osteopathic authors. As such, an examination of research endeavors held by allopathic and osteopathic neurosurgery residency applicants is warranted. The most recent data on charting outcomes for both ACGME and AOA residency applicants was published in 2014. According to the National Resident Matching Program (NRMP) 2014 Charting Outcomes, the mean number of research experiences and scholarly activities (i.e., abstracts, presentations, and publications) for US allopathic seniors matching into ACGME neurosurgery programs were 4.4 and 11.7, respectively [8]. The 2014 Osteopathic Graduate Medical Education Match Report reported a mean number of 3.3 research experiences and 6.9 scholarly activities for US osteopathic seniors matching into AOA neurosurgery programs [9]. Therefore, matched US osteopathic seniors had 1.1 less research experiences and 4.8 less scholarly activities than their allopathic counterparts in 2014. Just as distressing is the fact that US allopathic seniors who failed to match into an ACGME neurosurgery residency had more research experiences (3.7) and scholarly activities (8.4) than US osteopathic seniors who successfully matched into AOA neurosurgery programs [8]. We postulate that some of the many reasons why US osteopathic seniors applying for neurosurgery residency have substantially less research experiences and scholarly activities in the past compared to both matched and unmatched US allopathic seniors may be due to the relative lack of NIH funding, scholarly activity, and dual degree programs offered by osteopathic medical schools. Moreover, there has historically been a minimal emphasis placed on research experience and scholarly activities by osteopathic neurosurgical training programs. As such, the lack of research training in medical school and research experiences in residency may impede osteopathic neurosurgeons from gaining academic positions post-residency, and, ultimately, be causal factors for the apparent lack of proportionality between the percentages of US physicians and osteopathic neurosurgeons currently in practice and the authorship positions they hold in JNPG journals.

A major limitation of the current study is that only four neurosurgery journals were examined. The number of osteopathic physician authors may have been higher if additional journals were surveyed. Ideally, the percentage of osteopathic authors should be calculated with the number of American allopathic and osteopathic authors in the denominator. However, it was not feasible to look up the country of origin of 105,157 allopathic authors. All osteopathic physician authors are US-based but not all allopathic physician authors are. Thus, the total number of allopathic authors was counted without specifying American versus international origin. This may have made a slight difference in the percentage of osteopathic author representation in the journals. Nevertheless, JNSPG journals are American-based journals and the majority of authors’ country of origin is the US. Still, the validity of our results remains unchanged even if 90% of allopathic authors were found to be international allopathic physicians.

## Conclusions

In the present study, we observed a lack of proportionality between the percentages of practicing osteopathic physicians and osteopathic neurosurgeons and authorship positions they hold in JNPG journals. We provided several explanations for the disparity observed including remarkable differences between allopathic and osteopathic medical school NIH funding, research opportunities, and scholarly activities. The absence of a thriving research environment encountered in osteopathic medical schools may impede the development of academic physicians and, therefore, constrain osteopathic physicians from publishing during their careers. These data demonstrate that osteopathic medical schools are trailing far behind their allopathic counterparts in terms of research endeavors and manuscript authorship. With the ACGME-AOA merger in progress and residency spots becoming more competitive each year, osteopathic medical schools ought to correct this deficit for the sake of their graduating students and for the sake of their profession.

## References

[1] (2018). Association of American Medical Colleges 2017 State Physician Workforce Data Report. https://members.aamc.org/eweb/upload/2017%20State%20Physician%20Workforce%20Data%20Report.pdf.

[2] (2018). 1 in 4 new medical students enroll in a college of osteopathic medicine 125 years after founding of profession. https://www.osteopathic.org/inside-aoa/news-and-publications/media-center/2017-news-releases/Pages/9-19-one-in-four-new-medical-students-enroll-in-college-of-osteopathic-medicine-125-years-after-founding-of-profession.aspx.

[3] Ashurst JV, Galuska M (2016). Osteopathic physicians on the editorial boards of major medical journals over the past 30 years. J Am Osteopath Assoc.

[4] Hoehmann CL, Young CT, Fennie CN, Giniyani L, Nicholson CS, Collins JD, Cuoco JA (2017). Osteopathic physicians on editorial boards of neurosurgical journals: a quantitative analysis. J Spine Neurosurg.

[5] Clark BC, Blazyk J (2014). Research in the osteopathic medical profession: roadmap to recovery. J Am Osteopath Assoc.

[6] (2018). American Association of Colleges of Osteopathic Medicine 2016-2017 Academic Year Survey of Graduating Seniors Summary. http://AmericanAssociationofCollegesofOsteopathicMedicine2016-2017AcademicYearSurveyofGraduatingSeniorsSummary.

[7] Pheley A, Lois H, Strob J (2006). Interests in research electives among osteopathic medical students. J Am Osteopath Assoc.

[8] (2018). National Resident Matching Program Charting Outcomes in the Match. http://www.nrmp.org/wp-content/uploads/2014/09/Charting-Outcomes-2014-Final.pdf.

[9] (2018). Osteopathic Graduate Medical Education Match Report 2014. https://www.aacom.org/reports-programs-initiatives/aacom-reports/special-reports/ogme-match-2014.

